# Chitosan-Based Dressing as a Sustained Delivery System for Bioactive Cytokines

**DOI:** 10.3390/ijms25010030

**Published:** 2023-12-19

**Authors:** Sławomir Lewicki, Michał Zwoliński, Adrian Hovagimyan, Marta Stelmasiak, Łukasz Szarpak, Aneta Lewicka, Zygmunt Pojda, Łukasz Szymański

**Affiliations:** 1 Institute of Outcomes Research, Maria Sklodowska-Curie Medical Academy, 03-411 Warsaw, Poland; 2 Faculty of Medical Sciences and Health Sciences, Kazimierz Pulaski University of Radom, 26-600 Radom, Poland; 109274@student.uthrad.pl (M.Z.); 109352@student.uthrad.pl (A.H.); m.stelmasiak@uthrad.pl (M.S.); 3 Henry JN Taub Department of Emergency Medicine, Baylor College of Medicine, Houston, TX 77030, USA; lukasz.szarpak@uczelniamedyczna.com.pl; 4 Department of Clinical Research and Development, LUX MED Group, 02-676 Warsaw, Poland; 5 Military Centre of Preventive Medicine, 05-100 Nowy Dwór Mazowiecki, Poland; anet.lewicka@gmail.com; 6 Department of Regenerative Medicine, Maria Sklodowska-Curie National Research Institute of Oncology, 02-781 Warsaw, Poland; zygmunt.pojda@coi.pl; 7 Department of Molecular Biology, Institute of Genetics and Animal Biotechnology, Polish Academy of Sciences, 05-552 Magdalenka, Poland

**Keywords:** wound dressing, G-CSF, GM-CSF, stem cells, protein transfer to cells in vitro

## Abstract

Wounds represent a common occurrence in human life. Consequently, scientific investigations are underway to advance wound healing methodologies, with a notable focus on dressings imbued with biologically active compounds capable of orchestrating the wound microenvironment through meticulously regulated release mechanisms. Among these bioactive agents are cytokines, which, when administered to the wound milieu without appropriate protection, undergo rapid loss of their functional attributes. Within the context of this research, we present a method for fabricating dressings enriched with G-CSF (granulocyte colony-stimulating factor) or GM-CSF (granulocyte-macrophage colony-stimulating factor), showcasing both biological activity and protracted release dynamics. Based on Ligasano, a commercial polyurethane foam dressing, and chitosan crosslinked with TPP (sodium tripolyphosphate), these dressings are noncytotoxic and enable cytokine incorporation. The recovery of cytokines from dressings varied based on the dressing preparation and storage techniques (without modification, drying, freeze-drying followed by storage at 4 °C or freeze-drying followed by storage at 24 °C) and cytokine type. Generally, drying reduced cytokine levels and their bioactivity, especially with G-CSF. The recovery of G-CSF from unmodified dressings was lower compared to GM-CSF (60% vs. 80%). In summary, our freeze-drying approach enables the storage of G-CSF or GM-CSF enriched dressings at 24 °C with minimal cytokine loss, preserving their biological activity and thus enhancing future clinical availability.

## 1. Introduction

The healing of wounds is a complex biological process encompassing four interrelated stages: hemostasis, inflammation, proliferation, and remodeling [1]. Hemostasis, the initial phase lasting from minutes to hours, involves platelets preventing blood loss by triggering coagulation and clot formation. Platelet activation leads to the release of diverse cytokines, including CXCL4 (C-X-C motif chemokine ligand 4), hepatocyte growth factor (HGF), fibroblast growth factor (FGF), and transforming growth factor (TGF). These molecules modulate the behavior of local wound-residing cells and induce the release of chemokines that attract immune cells. CXCL4, notably, is a prominent chemokine during this stage, inhibiting angiogenesis while attracting T cells. Other chemokines, such as CXCL1, CXCL5, CXCL7, CLXCL8, CXCL12 (C-X-C motif chemokine, ligands: 1, 5, 7, 8, 10), CCL2, CCL3, and CCL5 (C-C motif chemokine, ligands: 2, 3, 5), are also released, albeit in smaller quantities [2,3]. Following the cessation of bleeding, the subsequent phase, inflammation, unfolds over 1 to 3 days. Damaged cells start this stage by releasing damage-associated molecular patterns (DAMPs) and reactive oxygen species (ROS), which locally activate immune cells. These immune cells then produce chemokines that entice further immune cells to the injury site [3]. Neutrophils and macrophages are the initial immune cells recruited to the wound. Their primary tasks encompass the removal of damaged cells, cellular debris, and pathogenic agents [4].

M1-like pro-inflammatory macrophages exhibit elevated secretion of matrix metalloproteinases (MMP): −1, −3, and −10. These enzymes contribute to the infiltration of compromised tissues by immune cells, which assume responsibility for microorganism elimination and inflammation maintenance. These M1 macrophages also play a pivotal role in effective wound healing by generating growth factors like TGF-β1 (transforming growth factor beta 1), FGF (fibroblast growth factor), PDGF (platelet derived growth factor), and VEGF (vascular endothelial growth factor) [5]. As the harmful signals (DAMPs and PAMPs—damage- and pathogen-associated molecular patterns) in the wound diminish, the activity of pro-inflammatory immune cells wanes, marking the onset of the third phase: the proliferation stage of wound healing. This phase, spanning a few days to a month, is characterized by the dominance of M2 macrophages and T regulatory cells. These entities orchestrate the proliferation of various cell types, including fibroblasts, keratinocytes, and endothelial cells. These cells, in turn, contribute to the formation of granulation tissue, re-epithelialization, and restoration of the vascular network. The regulatory cells oversee these processes by releasing growth factors, interleukins, and specific chemokines or directly activating neighboring cells through direct cell-to-cell interactions [6]. The final stage of wound healing, remodeling, unfolds from the 21st day up to a year after the initial injury. During this phase, collagen III is progressively replaced by the stronger collagen I. Ultimately, the scar attains full maturity [7].

Wound healing represents a multifaceted process governed by the orchestrated release of growth factors, interleukins, and chemokines in a temporal and spatial manner. Dysregulation in the distribution and timing of cytokine release can disrupt wound healing, leading to secondary infections, deformation of the wound site, or delayed recovery [8]. Optimal timing, location, and factor-specific release mechanisms hold the potential to significantly enhance treatment outcomes [9]. Researchers are in pursuit of ideal cytokine candidates that can expedite wound healing effectively and without adverse effects. Some cytokines, such as TGF-β and PDGF, are already undergoing clinical trials for this purpose [10,11]. In our study, we opted for two growth factors: G-CSF (granulocyte colony-stimulating factor) and GM-CSF (granulocyte-macrophage colony-stimulating factor), both known to augment wound healing processes. G-CSF is secreted by various cell types, including monocytes [12], endothelial cells [13], lymphocytes [14], and dermal fibroblasts [15]. It predominantly stimulates granulocyte production in the bone marrow, enhances mobilization of bone marrow-derived cells, and recruits them to injury sites [16]. Moreover, G-CSF stimulates keratinocyte growth in vitro [17]. GM-CSF, produced by both hematopoietic and non-hematopoietic cells, exhibits immunomodulatory properties. A pivotal effect of GM-CSF is the recruitment of macrophages, along with promoting proliferation and migration of keratinocytes and endothelial cells, culminating in wound closure and tissue remodeling [18]. Macrophages heavily rely on GM-CSF for optimal function, and its depletion results in impaired wound closure [19]. GM-CSF further stimulates the expression of peroxisome proliferator-activated receptors (PPARs), specifically PPAR-γ. Activation of PPAR-γ facilitates the transition of M1-like macrophages to M2-like macrophages, a shift that benefits wound healing and emerges as a potential target for chronic wound treatment [20]. Moreover, both cytokines exert influence on vascular endothelial cell proliferation (neoangiogenesis), keratinocytes, and fibroblasts [21]. Consequently, incorporating exogenous G-CSF and GM-CSF into wound healing therapies might restore immune equilibrium within the wound environment and enhance the overall healing process involving various cell types. A significant biological validation of these findings is exemplified in the study conducted by Tanha et al. In their investigation using a rat skin wound model, the authors demonstrated superior fibroblast maturation, increased collagen deposition, and a notable reduction in inflammation when employing a dressing composed of nanofibers containing G-CSF-loaded chitosan nanoparticles [22].

While integrating cytokines into wound healing therapies within clinical settings is relatively straightforward, implementing such therapies at home introduces certain challenges [23]. These include managing dressing manufacturing costs, where cost-effective production processes and materials must be balanced with quality standards. Administering bioactive molecules poses difficulties in designing user-friendly application methods, ensuring precise dosage control, and implementing monitoring mechanisms for optimal outcomes. Additionally, maintaining stability and shelf life of dressings in diverse home storage conditions necessitates addressing factors such as temperature variations and patient education on proper storage practices. Research in this field predominantly focuses on novel materials that incorporate bioactive molecules with immunomodulatory properties. As described in detail by Sousa at al., the wound healing process, being highly intricate, offers a wide array of bioactive molecules that can be used to facilitate the process [24]. Several studies involve utilizing anti-inflammatory cytokines and growth factors like VEGF and FGF. For instance, Chen et al. engineered electrospun poly(lactic acid) fibers loaded with IL (interleukin)-10, showcasing a cascade release pattern [25]. The initial IL-10 release curbed excessive inflammation, while subsequent releases maintained elevated IL-10 levels in the wound, facilitating macrophage polarization towards the M2 phenotype. Das et al. introduced an alginate hydrogel delivering syndecan-4 proteoliposomes (termed ‘syndesomes’) along with fibroblast growth factor-2 (FGF-2) to enhance wound healing [26]. This hydrogel exhibited immunomodulatory effects on wound macrophages, driving them towards the M2 phenotype and altering the cytokine profile. In a unique approach, Friedrich et al. explored the topical application of anti-TNF-α (tumor necrosis factor-α) combined with hyaluronic acid in a rat burn model. Their investigation revealed decreased macrophage infiltration and reduced IL-1β levels on the first day post-injury [27]. Xuan et al. devised a chitosan-silver hydrogel incorporating basic fibroblast growth factor for treating infected wounds [28]. This hydrogel effectively inhibited bacterial growth, promoted collagen deposition, and induced M2 macrophage polarization, thereby reducing the inflammatory response. Wang et al. reported the fabrication of a hydrogel comprising hyaluronic acid, dextran, and β-cyclodextrin loaded with resveratrol and a vascular endothelial growth factor plasmid [29]. When applied to wounds, this hydrogel attenuated the inflammatory response, leading to lower gene expression levels of IL-1β and TNF-α. Lastly, Yang et al. created a hyaluronic acid hydrogel incorporating extracellular vesicles derived from mesenchymal stem cells [30]. When applied to mouse skin injuries, this hydrogel directed macrophages towards the M2 anti-inflammatory phenotype. Innovative carriers offer the advantage of precise and controlled distribution and release of biologically active compounds. Nonetheless, the described experimental investigations, primarily characterized by their preliminary nature and absence of dedicated product development orientation, have yet to scrutinize the ramifications of storage conditions. Accordingly, our study focuses on developing suitable production methods for dressings containing G-CSF or GM-CSF cytokines. The goal is to ensure delayed cytokine release coupled with sustained biological activity.

## 2. Results

### 2.1. Effects of Different Preparation Techniques on Dressing

Chitosan crosslinked by sodium tripolyphosphate (TPP) was used to reduce the permeability of the dressing. Preliminary tests were conducted to ascertain the noncytotoxicity of dressing components alone (TTP or acetic acid) and optimize their composition concerning physicochemical parameters like fluid absorption and retention.

TPP at a concentration of 0.25% and acetic acid at 0.063% exhibited cytotoxic effects when tested as solutions not included in the Ligasano dressing (Figure S1). However, the incorporation of acetic acid at concentrations of 0.5% or 1% and TTP at concentrations of 0.25% or 0.5% during dressing preparation did not result in a substantial reduction in dressing cytotoxicity compared to 1% TTP and 2% acetic acid (Figure S2). Instead, it notably compromised the dressing’s physical properties, including absorbency and fluid retention. Therefore, the optimal formulation of the dressing was determined to be 2% acetic acid with 1% TTP. This formulation was established as mandatory for subsequent investigations. It was also determined during the production process that dialysis was necessary due to the dressing carrier’s cytotoxic properties.

**Figure 1:**
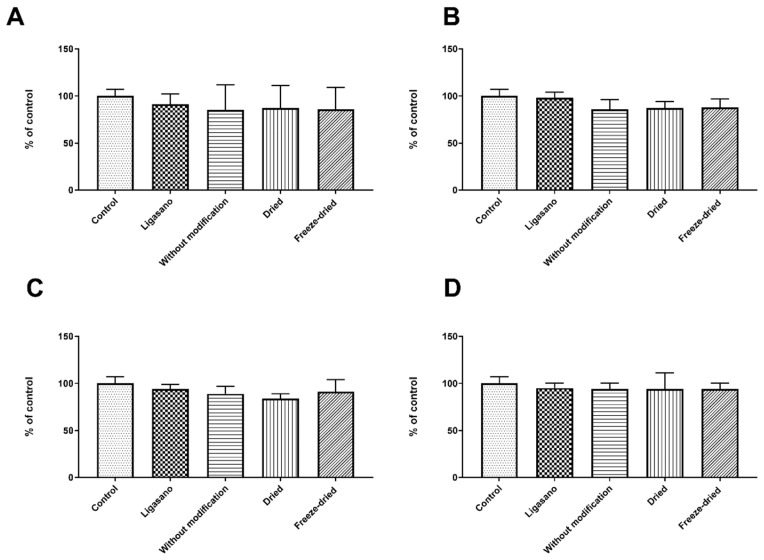
Effect of different dressing preparation techniques on cytotoxicity measured in UCSC cells. (**A**) Cell count; (**B**) MTT assay; (**C**) Neutral Red (NR) assay; (**D**) Sulforhodamine B (SRB) assay. Results are presented as mean ± standard deviation, *n* = 10 (n)—number of samples/group. There were no statistical differences between samples. Comparisons were made between experimental (Ligasano, without modification, dried, and freeze-dried) and control group values. Statistical analyses in all groups were performed by one-way ANOVA with Bonferroni correction.

The last step before adding growth factors to the dressing was to look at how the cytotoxicity of the optimal dressing changed depending on how it was made, how it was stored, and how long it was kept. Different dressing preparation techniques yielded statistically insignificant differences, irrespective of the technique employed for dressing preparation (drying or freeze-drying), across all cell count assessments conducted. Results are presented in Figure 1.

**Figure 2:**
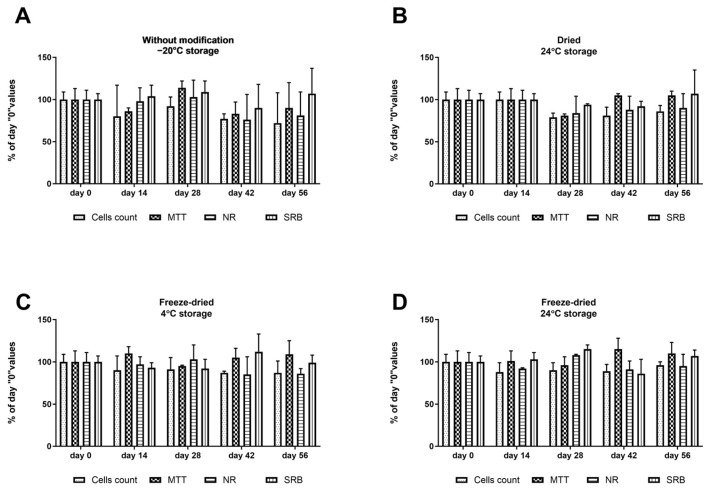
Effect of storage of differently dressing preparation techniques on cytotoxicity measured in UCSC cells. (**A**) Dressings without modification, −20 °C storage; (**B**) dried dressing, 24 °C storage; (**C**) freeze-dried dressing, 4 °C storage; (**D**) freeze-dried dressing, 24 °C storage. Cytotoxicity was measured immediately after production and after 14, 28, 42, and 56 days of storage in appropriate temperatures. Results are presented as mean ± standard deviation; *n* = 10 (n)—number of samples/group. There were no statistical differences between samples. Comparisons were made between experimental and control group values on day 0, 14, 28, 42, and 56. Statistical analyses in all groups were performed by one-way ANOVA with Bonferroni correction.

The growth of UCSC remains unaffected by the storage of “unloaded” dressings, regardless of the dressing type and storage conditions (Figure 2). This observation suggests that the sole determinant for the dressing preparation techniques will be the biological stability of cytokines and their release kinetics from the dressing.

### 2.2. Kinetics of Cytokine Release from the Dressing

**Figure 3:**
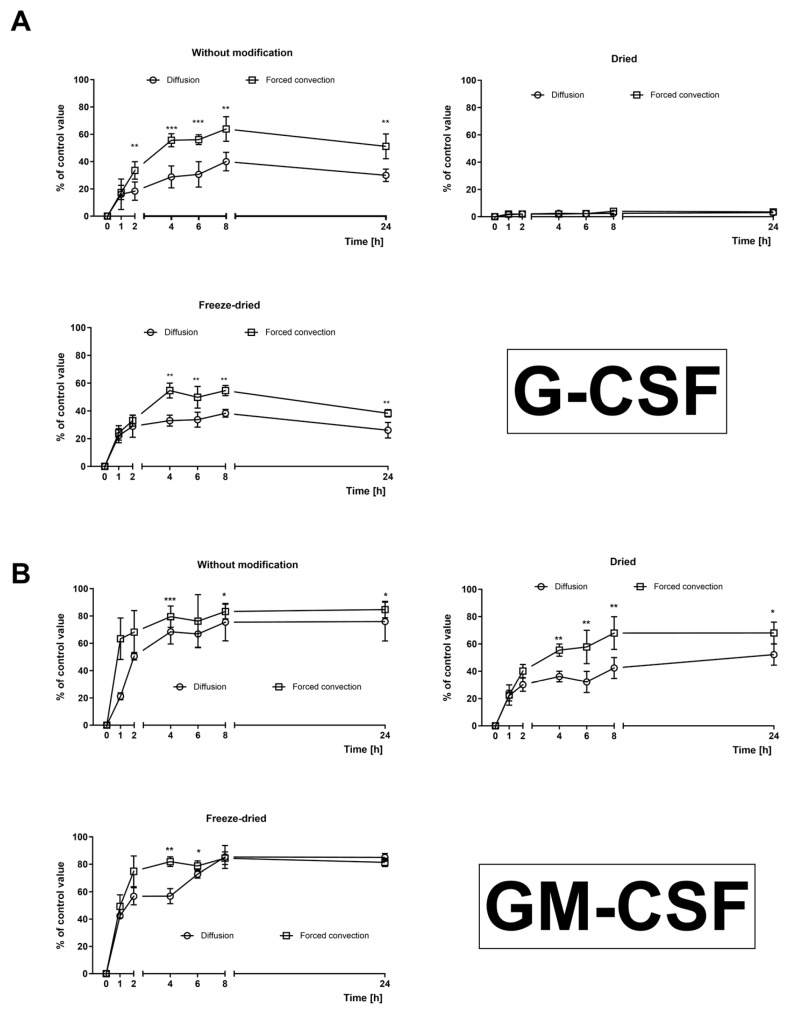
Cytokine release kinetics from dressings. Release of cytokines was performed in culture medium (Dulbecco Modified Eagle Medium, pH: 7.0–7.2, 4 °C) using diffusion and forced convection methods. (**A**) G-CSF release from unmodified, dried, or freeze-dried dressing; (**B**) GM-CSF release from unmodified, dried, or freeze-dried dressing. Results are presented as mean ± standard deviation and are expressed as a percentage relative to a control with cytokines matching the loaded drug concentration. *N* = 6 (n)—number of samples/group.*—*p* < 0.05; **—*p* < 0.01; ***—*p* < 0.001. Comparisons were between diffusion and forced convection at various times. Statistical analyses in all groups were performed by one-way ANOVA with Bonferroni correction.

The release kinetics of the growth factors from the dressing are intricately linked to the dressing preparation and storage techniques. G-CSF (Figure 3A) exhibits a notably slower and less efficient release into the environment compared to GM-CSF. In the context of simple diffusion, the process’s efficiency does not surpass 40%. The efficiency increases to 60% with forced convection. Interestingly, in the case of the dried dressing, there is no observable G-CSF release into the environment, regardless of the diffusion technique used.

For GM-CSF dressing (Figure 3B), the rate of cytokine release under simple diffusion is the most promising in the freeze-dried dressings. In this scenario, subsequent to the initial burst within the first hour, the release of the agent demonstrates an almost linear pattern over the ensuing seven hours, accounting for the release of approximately 80% of the loaded compound into the environment. Conversely, in the case of dried dressings, the release extends to 24 h but yields 20% less compound recovery compared to freeze-dried dressing. Dressings without modifications release the enclosed drug within the initial 4 h. Under forced convection (continuous medium mixing), across all variants, the drug release reaches its maximum within 4 h, converging with the final values achieved in simple diffusion.

### 2.3. Effect of Dressing Storage on Cytokine Recovery

**Figure 4:**
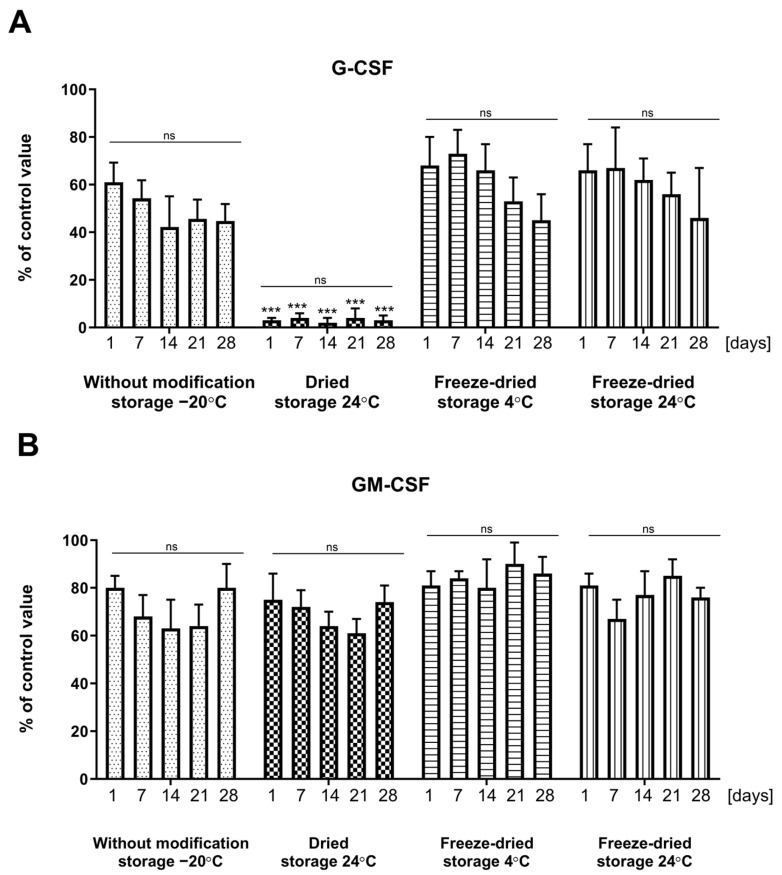
Effect of different preparation and storage techniques and storage (time and temperature) on the concentration of cytokines, measured in collected aliquots of the medium obtained in the cytokine recovery assay from the dressing, at 1, 7, 14, 21, and 28 storage days. (**A**) G-CSF; (**B**) GM-CSF. Results are presented as mean ± standard deviation and are expressed as a percentage relative to a control with cytokines matching the loaded drug concentration. *N* = 6 (n)—number of samples/group. Line—comparison within the production method group, ns—non significant, *** *p* < 0.001—comparison between each experimental group on the appropriate day of analysis. Statistical analyses in all groups were performed by non-parametric Kruskal-Wallis with Dunn’s multiple comparison test.

The investigation focused on the impact of dressing storage duration and conditions on the stability of loaded growth factors. Regarding G-CSF (Figure 4A), freeze-dried and dressings without modifications exhibit cytokine stability for at least one month. Nevertheless, the cytokine is recovered to a maximum of 60% when forced convection is used. In contrast, the dried dressing variant, consistent with previous findings, demonstrates complete ineffectiveness in this regard.

For dressings containing GM-CSF (Figure 4B), all dressing storage options ensure the chemical stability of the incorporated drug. In this context, the optimal choice appears to be dressings without modification or freeze-dried (stored at 4 °C or 24 °C), since it provides a high level of cytokine recovery, exceeding 80%.

### 2.4. Sterility Assessment and Cytotoxicity

The dressings, subsequent to their incubation in various culture media for a duration of one week, underwent microscopic examination following Giemsa and lactophenol cotton blue staining for bacterial and fungal detection, respectively. The analysis revealed an absence of observable fungi or bacteria within the culture fluid, affirming the sterility of the produced dressings.

**Figure 5:**
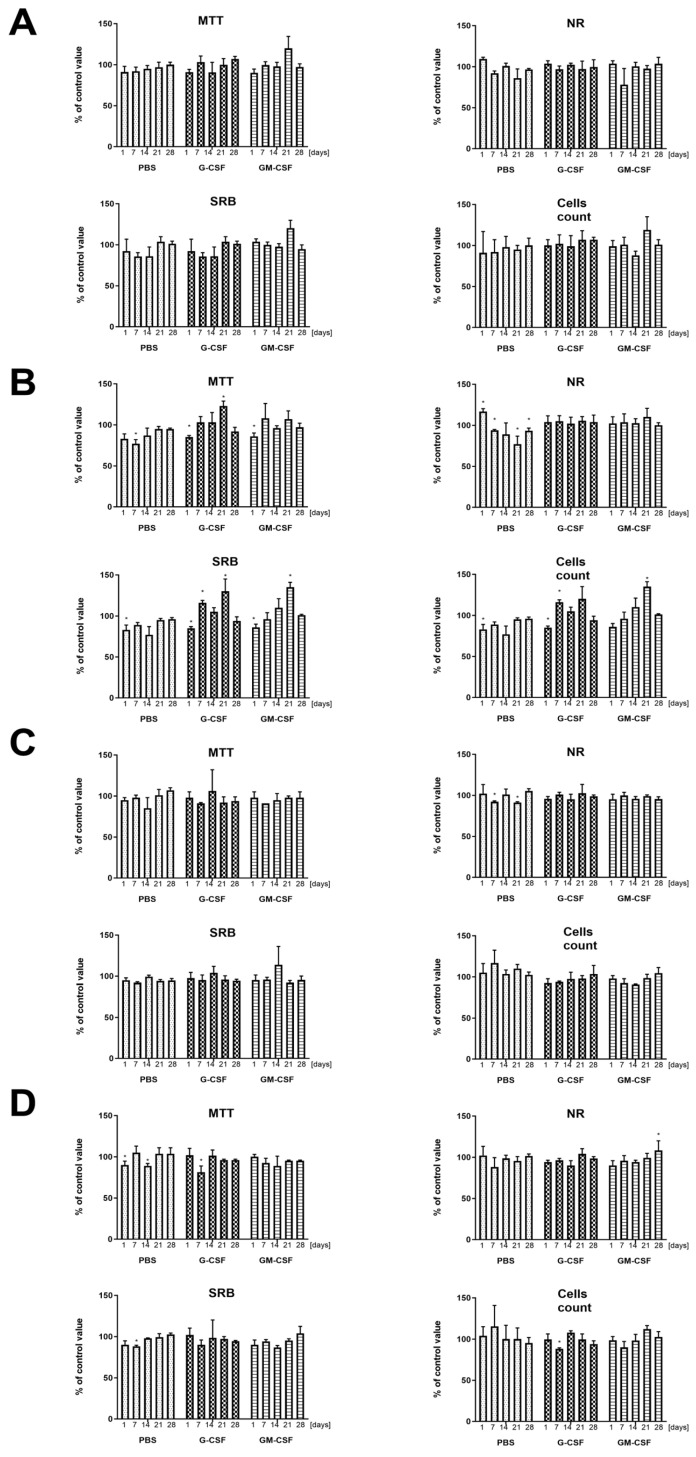
(**A**–**D**). Effect of different preparation and storage techniques and storage (time and temperature) on UCSC cytotoxicity. Cytotoxicity was measured by MTT, NR, SRB assays, and cell count after 1, 7, 14, 21, and 28 storage days. (**A**) Without modification, storage at −20 °C; (**B**) dried, storage at 24 °C; (**C**) freeze-dried, storage at 4 °C; (**D**) freeze-dried, storage at 24 °C. Results are presented as mean ± standard deviation and are expressed as a percentage relative to a control consisting of cells in culture medium without any additional agents. N = 10 (n)—number of samples/group. *—*p* < 0.05. The comparison was made between results obtained on the appropriate day of the experiment from the experimental groups (PBS, G-CSF, GM-CSF) and the control group (cells without any additional agents). Statistical analyses in all groups were performed by one-way ANOVA with Bonferroni correction.

**Figure 6:**
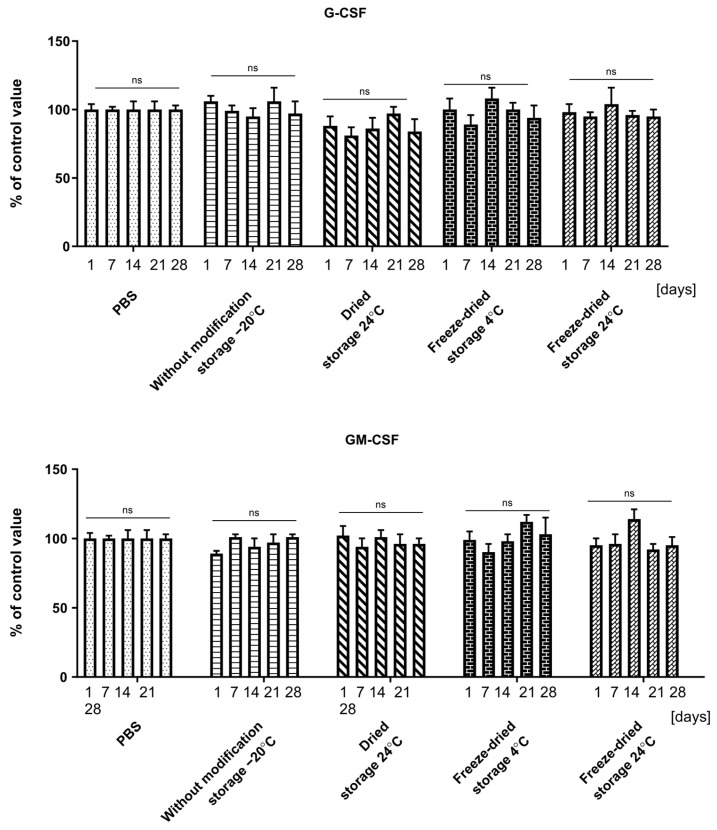
Effect of different preparation and storage techniques and storage (time and temperature) on granulocytes cytotoxicity. Cytotoxicity was measured using MTT-assay after 1, 7, 14, 21, and 28 storage days. Results are presented as mean ± standard deviation and are expressed as a percentage relative to a control consisting of cells in culture medium without any additional agents. *n* = 10 (n)—number of samples/group. Line—comparison within production method group, ns—non significant. There were no statistical differences between samples. Comparisons were made between experimental and control group values on day 0, 7, 14, 21, and 28. Statistical analyses in all groups were performed by one-way ANOVA with Bonferroni correction.

The performed assays determining the cytotoxicity of collected aliquots of the medium obtained in the cytokine recovery assay from evaluated dressings types generally showed no cytotoxic potential on UCSC or granulocytes. In some time-points and assay types, we found significant differences between control cells; however, these changes were not confirmed in all used tests. Results are presented in Figure 5A–D and Figure 6.

### 2.5. Biological Activity of Cytokines

**Figure 7:**
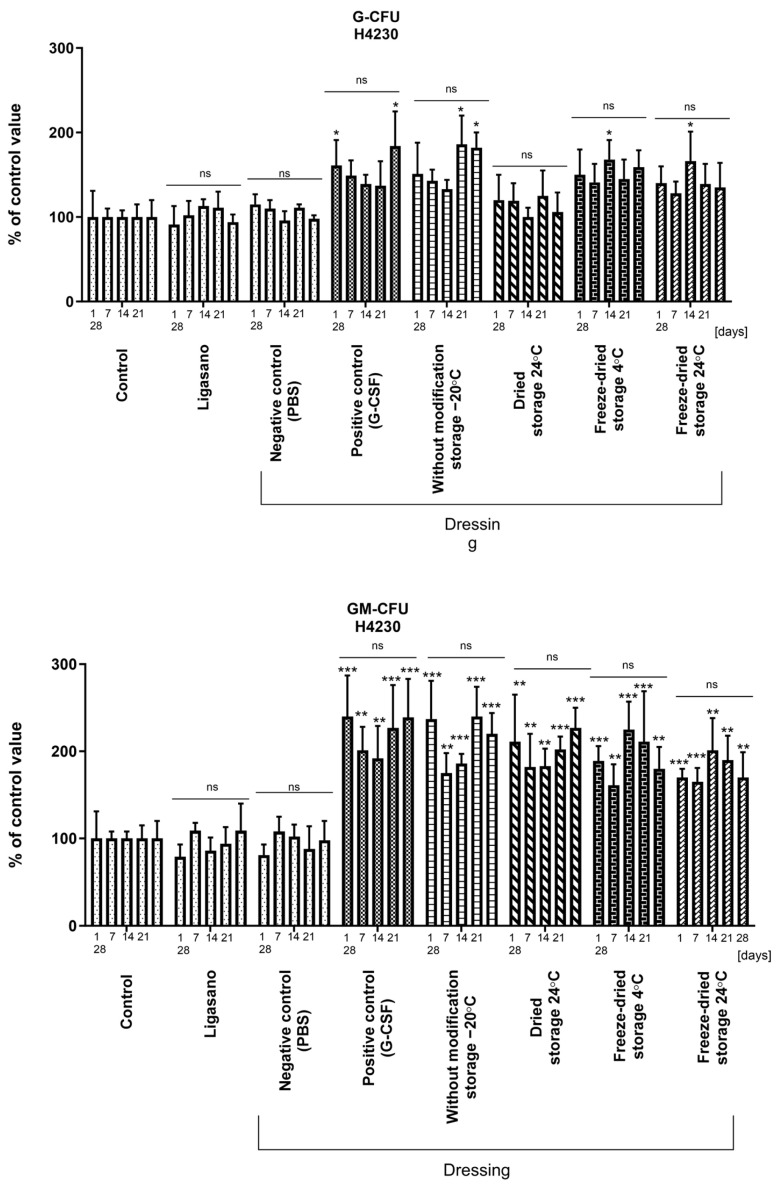
Evaluation of cytokines activity measured in the culture of cord blood hematopoietic stem cells in MethoCult™ H4230 methylcellulose medium without EPO. Cytokines were incorporated into dressings, which were prepared using different techniques and stored for 1, 7, 14, 21, and 28 days. Results are presented as mean ± standard deviation and are expressed as a percentage relative to a control with 50 µL of PBS containing 0.1% human albumin. *n* = 9 (n)—number of samples/group. Line—comparison within group, ns—non significant. *—*p* < 0.05; **—*p* < 0.01; ***—*p* < 0.001. Comparisons were made between experimental and control group values on appropriate days. Statistical analyses in all groups were performed using non-parametric Kruskal-Wallis with Dunn’s multiple comparison test.

Culturing cord blood hematopoietic stem cells (CBHSC) in a methylcellulose medium without EPO (erythropoietin) and growth factors (MethoCult™ H4230, Stem Cell Technologies, Vancouver, BC, Canada) exclusively led to their differentiation into granulocyte-macrophage cell colonies/cell forming units (GM-CFU). Findings from the assays evaluating hematopoietic precursors stimulated by collected aliquots of the medium obtained in the cytokine recovery assay from dressings containing growth factors reveal that the dressing preparation and storage methods ensure the preservation of the biological activity of the investigated cytokines within the dressings without modification and freeze-dried dressing groups (Figure 7). The count of colonies in these experimental groups remained comparable to that of the positive control, represented by a medium supplemented with freshly added cytokine. This phenomenon was observed for both G-CSF and GM-CSF, with GM-CSF exhibiting a more pronounced effect. Notably, the dried dressings variant with G-CSF (Figure 7) reiterated its inefficacy, yielding colony counts akin to those of control. Regarding the dried dressings with GM-CSF, the observed results were not significantly lower compared to other dressing preparation and storage variants.

The clonal growth assay employing culturing cord blood hematopoietic stem cells in a methylcellulose medium supplemented with EPO but lacking growth factors (MethoCult™ H4330) led to their differentiation into three colony types: mixed cells (Mix-CFU), erythroid cells (BFU-E), and granulocyte-macrophage cells (GM-CFU). No significant impact of dressing collected aliquots of the medium obtained in the cytokine recovery assay on erythroid cells was observed. However, the analysis of BFU-E, Mix-CFU, or GM-CFU colonies in this context presents inherent challenges and the potential for misinterpretation. This is particularly evident in MIX-CFU colonies that comprise both erythroid and granulocyte-macrophage cells, making their distinct separation practically impossible. Results of CBHSC growth in MethoCult™ H4330 medium are provided in Figure S3.

## 3. Discussion

Treating wounds, particularly extensive, chronic, or infected ones, poses a significant challenge to healthcare professionals. Consequently, researchers are continually devising methods and techniques to accelerate wound healing. Some of these techniques use specialized tools and/or strategies, which may need clinical settings, and therefore may limit their applicability. Hyperbaric oxygen therapy (HBOT) may be a good example. HBOT is primarily employed for radiation, burn, or diabetic wounds, enhancing short-term wound healing outcomes [31]. However, due to the high cost of the device and oxygen cylinder, its use at home is limited. Furthermore, it is even more challenging to use biological products, including adipose-derived stem cell (ADSC) cells, which require specialized equipment and laboratories but are known to accelerate the proliferation of epithelial cells, heighten collagen synthesis, and induce a transition from M1-like to M2-like macrophages [32]. In some techniques, such as negative pressure therapy (NPT), which has demonstrated efficacy in diabetic foot ulcers by expediting wound closure, reducing infection rates, and mitigating the risk of mortality [4], devices have been improved, making it possible to apply this method at home with promising results [33].

A prospective solution to this complex scenario could involve the development of dressings that offer both long-term, at-home utility and the inclusion of active agents that facilitate the wound healing process. Globally, researchers are investigating diverse compounds that hold the potential for advancing wound healing, with cytokines gaining considerable attention in this regard. An emerging tactic involves shifting the balance of wound healing from scarring towards regeneration through the application of TGF-β3. Unlike TGF-β1 and TGF-β2, TGF-β3 has demonstrated scar-suppressing properties and was shown to promote enhanced collagen arrangement in vivo [34]. Another noteworthy substance employed in wound healing is platelet-derived growth factor. Notably, patients with diabetic foot ulcers treated with PDGF exhibited a more significant reduction in percentage wound area than those subjected to standard treatment methods [35]. Our study concentrated on two specific growth factors, G-CSF and GM-CSF, which have shown promising effects in the wound healing process [36].

The initial step involves the identification of a suitable matrix for the integration of biological components. The adoption of carriers resolves the limitations inherent in cytokine-based therapies. Carriers obviate the need for frequent administration of rapidly inactivated growth factors, thus enabling a reduction in the cumulative daily dose and mitigating potential adverse effects [37]. Our study employed Ligasano (a commercial dressing) as the base for cytokine loading. To enhance the absorbency and biocompatibility of the carrier, we opted to incorporate chitosan. Chitosan, derived from chitin through deacetylation, serves a multifaceted role. Diverging from conventional dressings that merely envelop the wound, chitosan averts desiccation of the wound site and contributes to the healing process. Its effects vary depending on the phase of wound healing. During the hemostasis phase, chitosan expedites clot formation by attracting platelets. Subsequently, in the proliferation phase, it fosters cytokine release and supports tissue restoration. Within the proliferation phase, chitosan releases *N*-acetyl-D-glucosamine, which instigates fibroblast proliferation, augments collagen deposition, and promotes tissue remodeling. Eventually, during the maturation phase, *N*-acetyl-D-glucosamine moderates scar formation, thereby impacting the entire wound healing trajectory [38].

Chitosan is widely recognized as a highly advantageous material for bolstering the wound healing process. Its key merits include biodegradability, biocompatibility, non-toxicity, antimicrobial activity, biological functionality, adhesion capability, hemostatic influence, and water permeability [39,40]. The need for crosslinking in chitosan arises from its inherent structure, which lacks stability and durability for the aim of drug delivery [41]. These spaces enable facile fluid permeation without retention, prompting the requirement for crosslinking to modify this property. Extensive evidence substantiates that crosslinking augments resistance against acids and mechanical stress while facilitating controlled drug release by enhancing the material’s absorption capacity [42,43]. Chitosan may be crosslinked with ionic and covalent agents such as sodium citrate, TPP, sulfosuccinic acid, oxalic acid, glutaraldehyde, epichlorohydrin, trimethylpropane triglycidyl ether, or ethylene glycol diglycidyl ether [44]. In our study, crosslinking was accomplished using TPP and acetic acid. Chitosan’s amines undergo protonation in an acidic milieu, subsequently coagulating in the presence of anionic macromolecules. This process engenders a potential delivery mechanism for sustained therapeutic release. The optimal chitosan-to-TPP ratio emerged as a critical determinant in producing a resilient carrier. Additionally, the carrier’s biocompatibility was evaluated, with the most favorable absorption-to-cytotoxicity ratio achieved through a composition comprising 1% TPP and 2% acetic acid. The choice of ratio may be subject to variation based on the specific clinical context. For instance, a 4:1 chitosan-to-TPP ratio has demonstrated superiority in promoting mucin binding [45].

Ensuring the stability of a drug is a critical factor in its delivery, storage, and accessibility to patients. The cold chain system, requiring refrigeration throughout transportation, storage, and handling, imposes substantial economic and logistical challenges. This was especially visible during the SARS-CoV-2 pandemic [46]. For temperature-sensitive drugs, maintaining the cold chain is imperative to preserve their bioactivity. Any failure can result in the delivery of subtherapeutic doses or even render the product useless. Globally, the cost of maintaining the cold chain constitutes a significant portion of vaccination expenses [47]. Dry formulations offer substantial advantages over their liquid counterparts in this context. Several therapeutic agents have demonstrated enhanced thermostability over prolonged periods, thus mitigating stringent temperature requirements [48]. In our study, freeze-dried dressings exhibited stability comparable to unmodified storage at −20 °C for both G-CSF and GM-CSF. Dried dressings stored at 24 °C displayed elevated cytokine levels in the GM-CSF group, though not in the G-CSF group. In this context, the optimal approach appears to be utilizing freeze-dried dressings, regardless of the storage temperature. Unfortunately, we observed decrease in the cytokine concentration over time in G-CSF group for freeze-dried stored and at 4 °C and 24 °C. Therefore, our future work will focus on improving the G-CSF dressing stability.

During the subsequent step of our study, we directed our focus toward assessing the availability and kinetics of cytokine release from the dressings. Evidently, these attributes are intricately linked to the preparation and storage techniques employed. In this study, we demonstrate cytokine release assay data presented as a percentage in relation to a control incorporating cytokines matching the loaded drug concentration. This approach facilitates a more precise analysis of cytokine quantity, enabling direct comparison and accurate evaluation of the dressing’s total cytokine release over time. Specifically, the release profile of G-CSF proved notably slower and less efficient compared to that of GM-CSF. This observation aligns with the findings of Grzybowski et al., who explored cytokine release from dressings without modification, freeze-dried collagen, and polyurethane sponge in PBS at 37 °C. Within the freeze-dried group, the release of GM-CSF was higher than G-CSF, with values of 390.0 ± 136.0 ng (78% of the incorporated cytokine) and 16.0 ± 4.0 ng (3%), respectively [49]. In every instance, the cytokine concentrations failed to match those of the control (the 100% control value representing the loaded drug concentration), indicating that all evaluated conditions resulted in a reduction of the native cytokine concentrations. It should be noted, however, that our assay was performed at 4 °C. Furthermore, our choice of DMEM (Dulbecco’s Modified Eagle Medium) as a surrogate for the wound exudate, rather than the commonly utilized PBS in prior studies, was driven by the inclusion of compounds inherent to DMEM. These compounds encompass both inorganic elements (i.e., bicarbonate, calcium, magnesium, chloride, and phosphate salts) and organic constituents (i.e., amino acids or glucose), fostering an environment that more accurately simulates the composition of wound exudate [50,51]. The freeze-drying technique yielded the most promising results for GM-CSF, releasing over 80% of the compound. Additionally, G-CSF displayed greater instability, undergoing faster decay when compared to GM-CSF. However, it is imperative to articulate that the cytokine release assay performed in the current study is constrained by specific limitations. Primarily, the assay’s lower temperature may result in a decelerated cytokine release compared to the dynamics observed in a clinical setting. Conversely, the utilization of a DMEM-based exudate is just a limited model of accelerated cytokine release compare to the clinical scenario. Conversely, the use of a DMEM-based exudate model provides only a restricted representation, demonstrating an accelerated cytokine release in contrast to the complexity of the clinical scenario. This discrepancy is influenced by multifaceted factors, encompassing diverse wound characteristics such as size, depth, nature of damage, alongside exudate secretion volumes and its composition, encompassing proteins, lipids, saccharides, and various compounds contributing to the appropriate viscosity and pH within the wound milieu [52]. However, the most significant variance between our model and clinical observations lies in the volume applied in the cytokine release assay, notably exceeding the exudate volumes typically encountered in clinical settings [53,54]. To gain deeper insights into the impact of wound types and individual exudate compositions on the wound healing process, we advocate for comprehensive investigations undertaken by others [55,56,57]. Moreover, it is of significance to note that within our study model, we observed a bidirectional release of cytokines, where both sides of the dressing came into contact with the fluid. This stands in contrast to the unidirectional release typically observed in clinical settings, where only one side of the dressing interacts with the fluid. Chitosan proves to be a pore reducing filler for dressings, allowing for a substantial fraction of drug recovery after storage of previously freeze-dried dressings. The drug release kinetics’ may be influenced by the variations in molecular weights or deacetylation degrees among chitosan samples, along with the pH-dependent swelling characteristics of chitosan [58,59]. Under optimal conditions, chitosan hydrogels are capable of releasing over 80% of the drug within a single day [60], akin to the outcomes we observed for GM-CSF. What is more, Zaharoff et al. proved that chitosan enhances the GM-CSF immunoadjuvant properties by showing that chitosan solution maintained a measurable amount of recombinant GM-CSF at a subcutaneous injection site for up to 9 days in contrast to 12 to 24 h in case of saline [61]. In line with Zaharoff’s findings, Noh et al. showed that GM-CSF-loaded chitosan hydrogel increased the number of CD4+ and CD8+ INF-γ+ T cells, leading to enhanced humoral and cellular immunity [62]. Hydrogels based on chitin-alginate similarly exhibit a protracted pH-responsive drug release pattern. For instance, at 37 ± 0.5 °C and pH 7.4, 37% of metronidazole is released, while this value rises to 67% at pH 4.5, within a 24 h span [63]. Vakilian et al. study showed interesting patterns in a drug release system made of protein-loaded chitosan nanoparticles and poly-L-lactic acid (PLLA) hybrid nanofibers. A single-layer construct released 82% of the drug on the first day, while the multilayer configuration sustained the same drug release over 11 days at 37 ± 1 °C [64]. However, it should be noted that the molecules released in the studies referenced had markedly differing molecular masses, with some considerably lower (doxorubicin, metronidazole) or higher (BSA) than the cytokines utilized in the present investigation. Additionally, noteworthy variations in temperature conditions between these studies warrant consideration. Wound dressings designed for human application necessitate a noncytotoxic nature. Our selection of primary cell lines was guided by their heightened sensitivity to potential cytotoxic agents [65] than immortalized cell lines and the fact that the utility of using the standard cell line (mouse fibroblast L929) employed for cytotoxic evaluations in the registration of a medical device for cytotoxic evaluation is currently questioned [66]. Furthermore, mesenchymal stem cells, encompassing the UCSC cells employed in this investigation, exhibit a migratory propensity towards sites of tissue and organ injury, thereby orchestrating and overseeing the regenerative processes therein [67]. Moreover, other researchers, in their assessment of the impact of a novel nanofiber dressing incorporating cytokines like G-CSF, employed mesenchymal stem cells to assess the cytotoxicity of the developed product [22]. A negative effect of a dressing on those cells may translate to an unfavorable influence on the wound healing process. Due to both reasons, we used UCSC cells to test the cytotoxic potential of our dressings. Through our assays evaluating the cytotoxicity of collected aliquots of the medium obtained in the cytokine recovery assay derived from the investigated dressings, we discerned no adverse impact on UCSC cells or granulocytes in a cultured environment. Consistent with existing research, chitosan is generally well-tolerated by cells [68], particularly when employing low molecular weight variants [69]. However, in our study, chitosan, crosslinked with TPP in acetic acid, did manifest some cytotoxic potential, as both components exhibited cytotoxic effects in UCSC cells (Figures S1 and S2). Consequently, in the formulation of the current wound dressing, we implemented a 12 h dialysis procedure in distilled water, effectively mitigating its cytotoxicity. Notably, the incorporation of cytokines (G-CSF or GM-CSF) did not introduce any alterations in the cytotoxicity potential of the dressing.

The final but most significant stage in the production of wound dressings incorporating cytokines involves assessing their biological efficacy. For this, we used primary umbilical cord blood hematopoietic stem cells, which respond well to the addition of G-CSF or GM-CSF by increasing the clonal growth of granulocyte and macrophage precursors both in vitro and in vivo [70]. When cultured in methylcellulose without erythropoietin, these stem cells exclusively differentiate into granulocyte-macrophage cell colonies (GM-CFU). The dressings without modification group, freeze-dried dressings stored at 4 °C and 24 °C, and the dried dressing group all yielded GM-CFU levels equivalent to those of the positive control. The impact of these dressings resembled that of soluble GM-CSF. In the case of G-CSF, all dressing types except those stored in dry conditions achieved levels comparable to the positive control; however, the overall effect was lesser compared to GM-CSF. Based on these results, freeze-drying seems to be the preferable option for dressing production due to the fact that the dressing can be stored at room temperature (24 °C). This facilitates its use in both clinical and home settings. In the presence of erythropoietin-containing medium, the cells differentiated into three colony types: mixed cells (Mix-CFU), erythroid cells (BFU-E), and granulocyte-macrophage cells (GM-CFU). In summary, GM-CSF outperformed G-CSF, particularly in the generation of GM-CFU, yet the treatment groups exhibited considerable result dispersion. Our findings align with existing research. Yuan and Liu [71] formulated a hemostatic gauze scaffold infused with G-CSF, achieving loading efficiency exceeding 95% and only a minimal decrease in G-CSF content in dressings compared to the solution. Release of the contents transpired over time, depending on the preparation method, reaching a plateau within 5 to 8 days, with cumulative release spanning from 30% to 95%. Their study revealed an extended elevation in neutrophil levels in the group receiving more G-CSF over an extended duration. Likewise, Huang et al. explored the use of GM-CSF with alginate dressing for refractory chronic skin ulcers. Their research demonstrated that the combination of alginate and GM-CSF accelerated the healing rate and reduced pain intensity [72]. Similar findings were reported by Jaschke et al., where a compressive dressing coupled with GM-CSF achieved a 90% healing rate and prevented ulcer recurrence [73]. Moreover, Salva et al. showed that chitosan/pGM-CSF complexes accelerated wound healing in the early and late phases in vivo [74]. Dehkordi et al. also showed the potential of GM-CSF chitosan complexes. In their work, the wounds covered with GM-CSF loaded chitosan nanoparticles achieved full closure and complete re-epithelialization after 13 days, compared to the normal saline treated wounds, which exhibited nearly 70% of wound size reduction [75]. These results further confirm the potential translational applicability of described GM-CSF dressings.

## 4. Materials and Methods

### 4.1. Cytokines

Lyophilized G-CSF and GM-CSF cytokines were purchased from Peprotech IMC (Cranbury, NJ, USA). Cytokines were dissolved in phosphate-buffered saline (PBS, Gibco, Warsaw, Poland) with 0.1% human albumin (Octapharma, Warsaw, Poland) in a 100 ng/mL concentration, aliquoted, and stored at −80 °C until use.

### 4.2. Dressing Carrier Fabrication

As a base for dressing development, we used commercially available sterile Ligasano dressings, with dimensions of 10 cm × 10 cm × 1 cm (Ligamed, Cadolzburg, Germany). Given that Ligasano has relatively large pores, we decided to enhance its structure by introducing chitosan crosslinked by TPP. Chitosan (Sigma Aldrich, Poznan, Poland) was dissolved in 2% acetic acid (Sigma Aldrich, Poland) at a ratio of 1 g of chitosan per 50 mL of 2% acetic acid. The solution was agitated on a magnetic stirrer with heating (100 RPM, 40–50 °C) for 1 h. Ligasano was introduced into a sterile Petri dish, followed by the addition of dissolved chitosan (40 mL). Employing a sterile bacterial spreader, the solution underwent meticulous application onto the dressing, undergoing a 5 min duration of pressurized treatment to ascertain complete saturation of Ligasano with chitosan. Subsequently, the dressing was immersed in 400 mL of a 1% TPP solution in distilled water (Sigma Aldrich, Poland) for an hour at room temperature. The dressing was flipped every 15 min during this incubation. Following the 1 h TPP treatment at room temperature, the dressing was blotted using sterile gauze and subjected to 2 rounds of 30 min incubation in 1 L of distilled water on a magnetic stirrer (10 RPM (revolutions per minute), temperature 4 °C), with each cycle of incubation followed by drying on sterile gauze. Despite the enhanced absorption parameters, these dressings exhibited signs of cytotoxicity. To address this, dialysis was performed over 12 h at 4 °C, involving the addition of 3.5 L of sterile distilled water for each 10 × 10 cm dressing. The dressings were dried again using sterile gauzes over 48 h at 56 °C. Finally, the dressings were sectioned into fragments measuring 1 × 1 cm. These prepared dressings served as the foundation for subsequent research. All stages of dressing fabrication were executed under antiseptic conditions within a Class II laminar flow chamber.

### 4.3. Incorporation of Cytokines into Dressing Carrier

Each dressing carrier (1 cm × 1 cm) was impregnated with 100 µL of a solution: PBS with 0.1% human albumin as the control, or a 100 µL solution of G-CSF or GM-CSF (Peprotech IMC) at a concentration of 20 μg/mL (equivalent to 2 μg per dressing) in PBS, supplemented with 0.1% human albumin. Subsequently, the dressings were categorized into three groups: 1. Not subjected to further procedures, labeled “without modification”; 2. Exposed to a drying process (37 °C, 24 h “dried”); and 3. Subjected to freeze-drying. Following these treatments, the dressings were either subjected to analysis or preserved at different temperatures, aligning with potential storage conditions for widespread applications.

### 4.4. Kinetics of Cytokine Release from the Dressing

To study the release of cytokines, G-CSF or GM-CSF dressings were put in a 6-well dish with 10 mL of DMEM culture medium and 0.1% human albumin. Collection of aliquots of the medium with dressings (cytokine drug release assay) was performed at 4 °C over a period of 1 to 24 h using two distinct methods: (1) forced convection, employing a plate shaker (75 RPM) and (2) normal diffusion. At time intervals of 1, 2, 4, 6, 8, and 24 h, the samples (one sample was taken from three independent technical replicates, 50 μL each, and mixed) were collected, and concentrations of cytokines were assessed via ELISA (R&D Systems, Minneapolis, MN, USA), following the manufacturer’s instructions. Subtracted aliquots of the medium were not replaced by fresh medium. Each sample for ELISA analysis was subtracted from five independent experiments. As a control, a culture medium (10 mL) supplemented with 100 μL of cytokines equal to the concentration of the loaded drug onto the dressing was used to account for cytokine interactions with the test system. The cytokine release results are presented as a percentage in relation to this control.

### 4.5. Effect of Storage Conditions on Cytokine Recovery

Four experimental groups were established:

1. Dressings without modification: Dressings containing cytokines were placed in hermetically sealed containers, frozen at −80 °C, and preserved until analysis;
2. Dried dressings: Dressings with cytokines were dried at 37 °C for 24 h, placed in hermetically sealed containers, and stored at room temperature in the dark;
3. Freeze-dried dressings (4 °C): Dressings containing cytokines were lyophilized, placed in hermetically sealed containers, and stored at 4 °C;
4. Freeze-dried dressings (24 °C): Dressings with cytokines were lyophilized, placed in hermetically sealed containers, and stored at 24 °C.

Quantitative analysis of cytokines recovery from the stored dressings was conducted at specific time intervals, specifically 1, 7, 14, 21, and 28 days. During each time point, dressings were individually transferred to culture medium-filled dishes (10 mL each) and subjected to a 4 h forced convection process (selected based on prior analysis as yielding optimal outcomes from collected aliquots of the medium obtained in the cytokine release assay). The collected aliquots of the medium obtained from this procedure (one sample was taken from three independent technical replicates, 50 μL each, and mixed) were utilized for both quantitative cytokine recovery analysis through ELISA tests and biological evaluations. An independent sample and its replicates were employed for each storage time. A control group was established using 4 h incubation of appropriate cytokines (added concentration 2 μg) from a stock solution in a culture medium (10 mL). The cytokine recovery results are presented as a percentage in relation to control.

### 4.6. G-CSF and GM-CSF Concentration Analysis

The concentration of G-CSF or GM-CSF in all samples was evaluated using an ELISA kit (R&D Systems, USA, Human G-CSF Quantikine ELISA Kit and Human GM-CSF Quantikine ELISA Kit), following the manufacturer’s instructions. To ensure measurements within the detection range of the ELISA kits, GM-CSF samples were diluted at a 1:5 ratio using the dilution buffer provided with the ELISA kit. Each sample was analyzed in duplicate during the ELISA analysis.

### 4.7. Cell Isolation, Identification, and Culture

To ensure the noncytotoxic nature of the dressings and assess the retained biological activity of the incorporated cytokines, three distinct primary cell lines were employed: umbilical cord stem cells (UCSC), cord blood hematopoietic stem cells (CBHSC), and granulocytes. These cells were isolated from healthy volunteers, with the protocol approved by the Ethical Committee under permit no. KB 70/2012.

#### 4.7.1. Umbilical Cord Stem Cells (UCSC)

UCSC were isolated as previously described [76]. Briefly, umbilical cord fragments were isolated after childbirth (Ethical Committee, permit no. KB 70/2012). Fragments of Wharton jelly were transferred to the culture flask (75 cm^2^, Corning Life Sciences, Warsaw, Poland) and maintained in growth medium (DMEM with GlutMax, 20% of FBS, and 50 UI/mL of antibiotics: penicillin/streptomycin; all from Gibco, Poland). Cells were passaged 3–5 times before being utilized. After the 3rd passage, the cells underwent phenotyping to determine the presence of mesenchymal stem cell markers CD29, CD34, CD45, CD73, CD90, CD105, and CD106 (all antibodies were procured from Becton Dickinson, Warsaw, Poland). The analysis of all markers was performed using flow cytometry (FACS Calibur, Becton Dickinson, Warsaw, Poland). Further investigations were carried out using cells (95% of isolated cells) exhibiting high expression of CD29, CD90, CD73, and CD105, weak expression of CD106, and the absence of CD34 or CD45 markers.

#### 4.7.2. Cord Blood Hematopoietic Stem Cells (CBHSC)

CBHSC were isolated as previously described [76]. Cord blood samples were collected during delivery with the mothers’ consent. The mononuclear cells were isolated by Ficoll–Uropoline centrifugation 400× *g*, 40 min (Stem Cell Technologies, Vancouver, BC, Canada) and frozen in liquid nitrogen (−170 °C) until further use.

#### 4.7.3. Granulocytes

Granulocytes were isolated from the peripheral blood of healthy volunteer donors. Blood was collected in a volume of 20 mL into syringes containing 200 units of heparin (Polfa, Warsaw, Poland) as an anticoagulant. After 1:1 dilution with PBS, blood was applied to 10 mL of Gradisol G (Polfa, Warsaw, Poland) in a 50 mL tube and centrifuged for 30 min (400× *g*, room temperature). Subsequently, the lower interphase containing granulocytes was extracted and rinsed three times with PBS. The cells were resuspended in IMDM medium (Gibco, Warsaw, Poland) and used immediately for analysis. The purity of the isolated cells was assessed based on flow cytometry parameters (FSC/SSC) and CD66b expression.

### 4.8. Biological Assays

#### 4.8.1. Preparation of Collected Aliquots of the Medium Obtained in the Cytokine Drug Release Assay for Biological Evaluation

For the biological assessment of cytokines, we employed a 4 h forced convection method in culture medium, as it yielded the most optimal cytokine recovery results. The collection procedure used was consistent across ELISA, cytotoxicity, and CBHSC analysis. Following a 4 h convection of the dressing, the medium (collected aliquots) was harvested and promptly subjected to ELISA, cytotoxicity, and CBHSC assays.

#### 4.8.2. Cytotoxicity

Tests were performed on UCSC cells or granulocytes. The preliminary evaluation of the cytotoxicity of TPP and acetic acid, independent of Ligasano, was conducted using cell counts and MTT (3-[4,5-dimethylthiazol-2-yl]-2,5-diphenyltetrazolium bromide; thiazolyl blue) assays with UCSC cells (umbilical cord stem cells).

Four distinct assays, cell number count using a Bürker chamber, MTT-assay [77], NR uptake assay (NR—neutral red) [78], and SRB-assay (SRB—sulforhodamine B) [79], were employed to assess the evolution of dressing cytotoxicity in UCSC cells, as these represent the predominant isolated adherent cell population with proliferative capabilities, rendering them a reliable indicator for determining the cytotoxic or cytostatic nature of the evaluated factor. In contrast, in vitro cultured blood granulocytes lack proliferative capacity, limiting their utility to evaluate only pronounced toxicity. Consequently, the widely adopted MTT assay was utilized to perform this assessment, given its suitability for appraising cytotoxic effects.

During the logarithmic growth phase, UCSC cells were trypsinized and centrifuged (5 min, 400× *g*). The cells were then seeded onto a 24-well culture plate at 2 × 10^4^ cells per well. Following 24 h of incubation, the growth medium was removed, and collected aliquots of the medium obtained from the appropriate dressing were introduced into the wells. After an additional 24 h, assays were conducted for assessing dressing cytotoxicity. Data were collected from five independent experiments with a sample size of 10. The results are expressed as percentages of the control value (cells without any additional agents) ± standard deviation.

After blood isolation, granulocytes were resuspended in IMDM medium (Gibco, Warsaw, Poland) with 10% FCS (Gibco, Warsaw, Poland), and seeded at a density of 1 × 10^6^ cells per well in a 12-well plate. The cells were then centrifuged, and aliquots of the medium obtained from dressings were added to the cells. After 24 h of incubation, the MTT test was conducted. The data were acquired from 5 independent experiments, with a sample size of 10. The outcomes are presented as percentages of the control value (cells without any additional agents) ± standard deviation.

#### 4.8.3. CBHSC Assay

Clonal growth assay of hematopoietic cells (CBHSC) was used to test the biological activity of dressings with G-CSF or GM-CSF. CBHSC cells were cultured in 24-well plates, with each well containing 1 × 10^4^ cells. A commercial methylcellulose medium was utilized for the culture, and two variants of this medium were employed, both from Stem Cell Technologies: MethoCult™ H4230 without growth factors and without EPO, and MethoCult™ H4330 without growth factors but with EPO. Prior to introducing the cells, 50 μL of collected aliquots of the medium obtained in the cytokine recovery assay from the dressing was extensively mixed with 950 μL of the respective methylcellulose medium. The concentration of cytokines was determined prior to adding the collected aliquots of the medium obtained in the cytokine recovery assay. Approximately 10 ng/mL of G-CSF or GM-CSF (50 μL) was added for each experiment. The positive control was composed of 50 µL of pure cytokines in PBS with 0.1% human albumin, maintaining a 10 ng/mL concentration. The control group consisted of 50 µL of PBS with 0.1% human albumin (without cytokines). A volume of 50 µL of the suspensions from Ligasano and dressing developed with PBS instead of cytokines (negative control) were also evaluated. The number of cells, including mixed cells (Mix-CFCs), erythroid cells (BFU-Es), or granulocyte-macrophage cells (GM-CFCs), was evaluated after 14 days of culture. Colonies were identified and counted directly under an inverted microscope (10× objective) by two independent researchers. The distinction between CFUs was made according manufacturer procedures. Obtained results were then averaged, and further statistical analyses were performed. The results are presented as percentages relative to control cells after 14 days of culture (mean ± standard deviation). All experimental procedures were conducted in triplicate, n = 9.

#### 4.8.4. Sterility Assessment

The prepared dressings were transferred to 6-well plates, and 10 mL of culture medium (DMEM, RPMI, or alpha-MEM + 10% FBS (fetal bovine serum), all from Gibco, Poland) was added. The plate with the dressings was then positioned inside a cell culture incubator (maintained at 37 °C with 5% CO_2_) and left for a duration of one week. Following this week, the dressings were taken out, and the culture fluid was examined by Giemsa staining for the detection of bacteria and lactophenol cotton blue staining for fungi detection. After staining, the fluid was examined under a microscope to detect any presence of microorganisms (100× and 1000× magnification).

### 4.9. Statistical Analysis

Statistical evaluation of the results was performed using one-way ANOVA with Bonferroni correction (in the case of a normal distribution) or non-parametric Kruskal–Wallis with Dunn’s multiple comparison test (in the case of a non-Gaussian distribution). The distribution of the data was evaluated using the Shapiro–Wilk test. GraphPad Prism software was used to carry out these tests (version 7; GraphPad Software, Inc., La Jolla, CA, USA). *p* < 0.05 was considered a statistically significant difference.

## 5. Conclusions

Chitosan-based dressings, developed and evaluated in this work, exhibit substantial promise as carriers and allow the loading and release of bioactive molecules with dimensions much higher than those of typical drug molecules. These dressings exhibit promise for wound healing applications owing to their protracted cytokine release profile. Furthermore, even after storage, the cytokines extracted from these dressings preserved their biological activity. However, additional validation remains imperative, considering the disparity between these conditions and those within the wound bed. Our investigation underscores freeze-drying followed by room temperature storage as a viable approach with a commendable safety profile. This method demonstrates negligible cytokine loss, offering positive outcomes in in vitro tests assessing cell proliferation and differentiation. Furthermore, the potential for freeze-dried dressings to be stored under more relaxed temperature conditions may significantly improve their clinical accessibility. Nonetheless, comprehensive research remains imperative to scrutinize the efficacy and safety of wound dressings and incorporate growth factors in an in vivo model.

## Data Availability

The data presented in this study are available on request from the corresponding author. The data are not publicly available due to founding agreement limitations.

## Supplementary Materials

The supporting information can be downloaded at: https://www.mdpi.com/article/10.3390/ijms25010030/s1.
