# Supplementary material for: Chitosan-Based Dressing as a Sustained Delivery System for Bioactive Cytokines
Source: Int J Mol Sci. 2023 Dec 19;25(1):30. doi: 10.3390/ijms25010030 (PMC10778940; doi:10.3390/ijms25010030)
Supplement: Supplementary file 1 [file ijms-25-00030-s001.zip › ijms-2625193-supplementary.pdf]

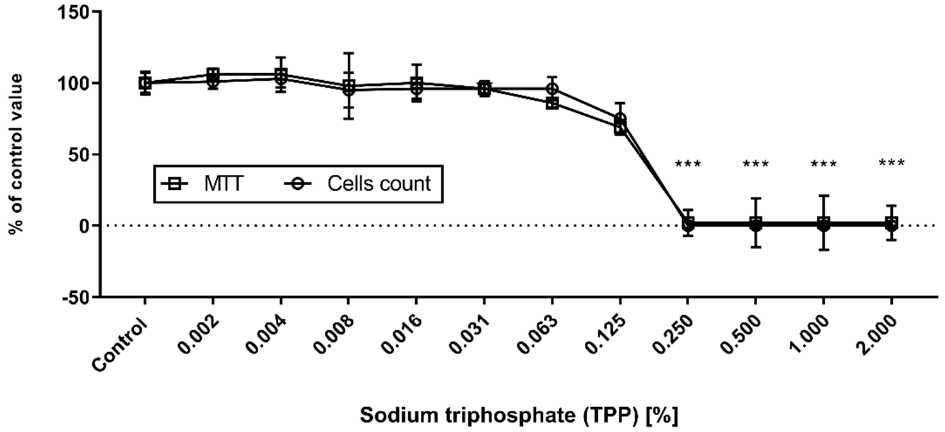

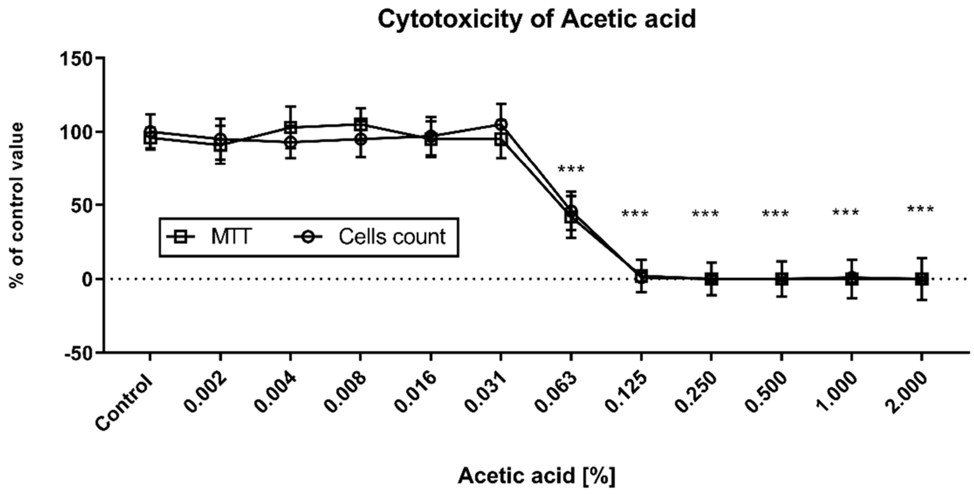

Figure S1. Effect of TPP or acetic acid on cytotoxicity on UCSC cells (both compounds were tested as solutions not included in the Ligasano dressing). Results are presented as mean ± standard deviation and are expressed as a percentage relative to a control consisting of UCSC cells in culture medium without any additional agents. n = 10 (n) - number of samples/group. *** - $p < 0.001$ (compared to control values). Statistical evaluations were performed between the experimental and control group. Statistical analysis in all groups was performed by one-way ANOVA with Bonferroni correction.

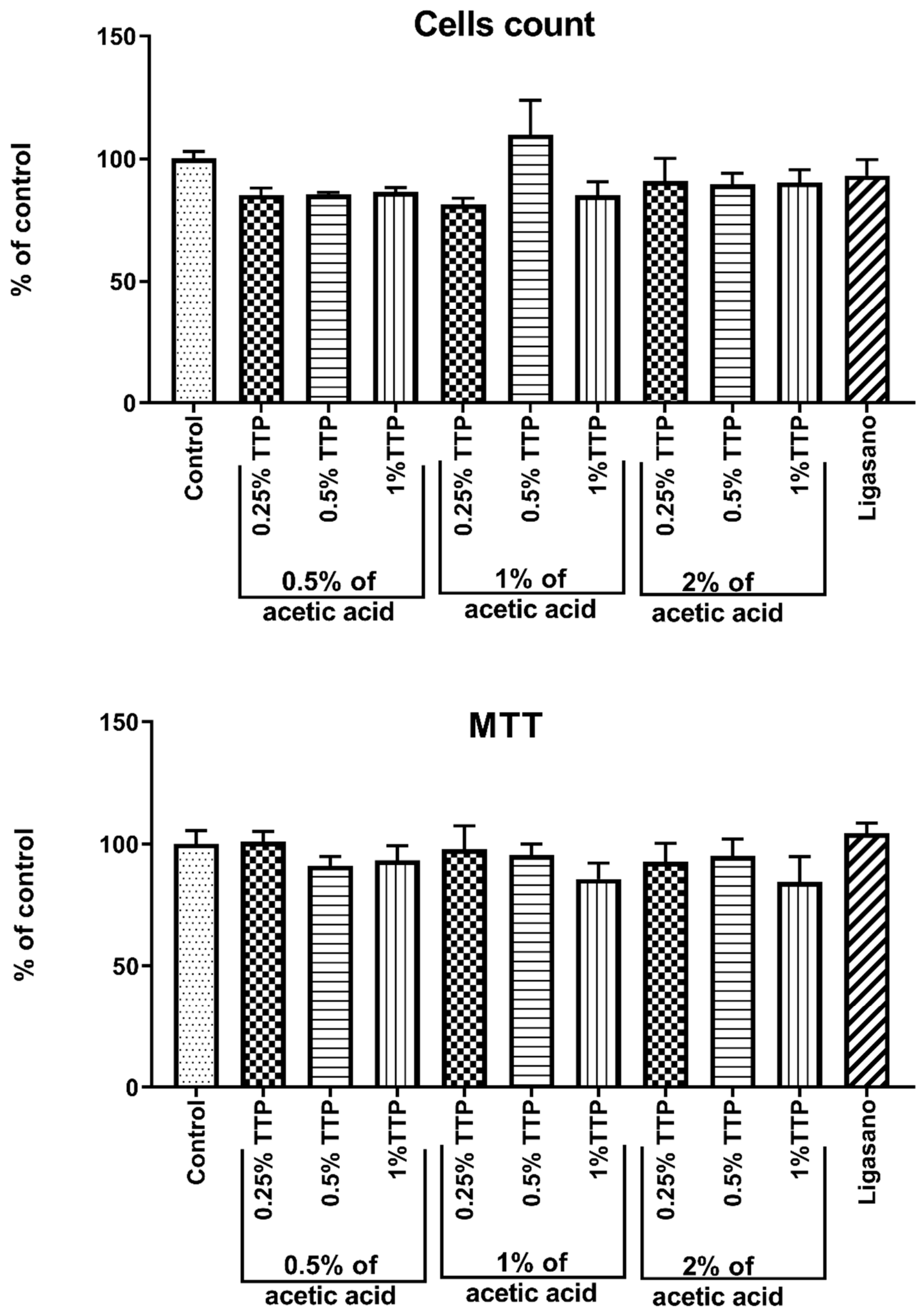

Figure S2. The impact of varying concentrations of TPP and acetic acid employed in dressing preparation and storage techniques on the dressing cytotoxicity on UCSC cells. Results are presented as mean ± standard deviation and are expressed as a percentage relative to a control consisting of UCSC cells in culture medium without any additional agents. n = 10 (n) - number of samples/group. There were no statistical differences between samples. Statistical evaluations were performed between the experimental and control groups. Statistical analysis in all groups was performed by one-way ANOVA with Bonferroni correction.

A

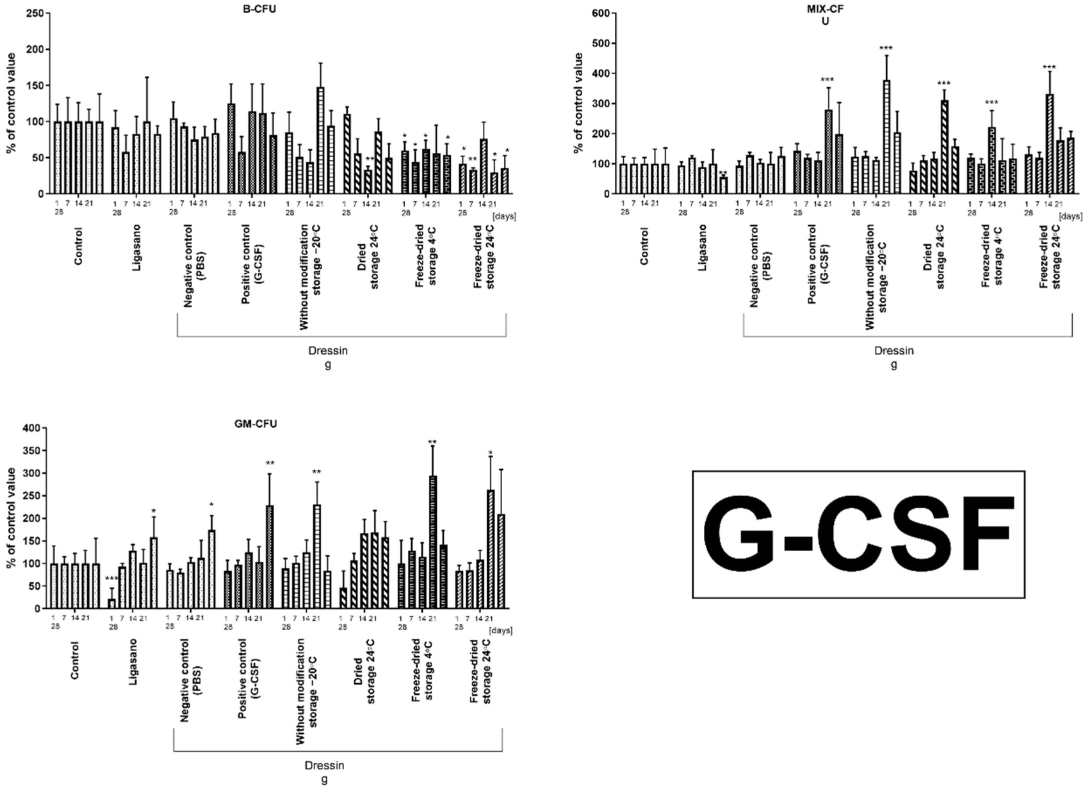

B

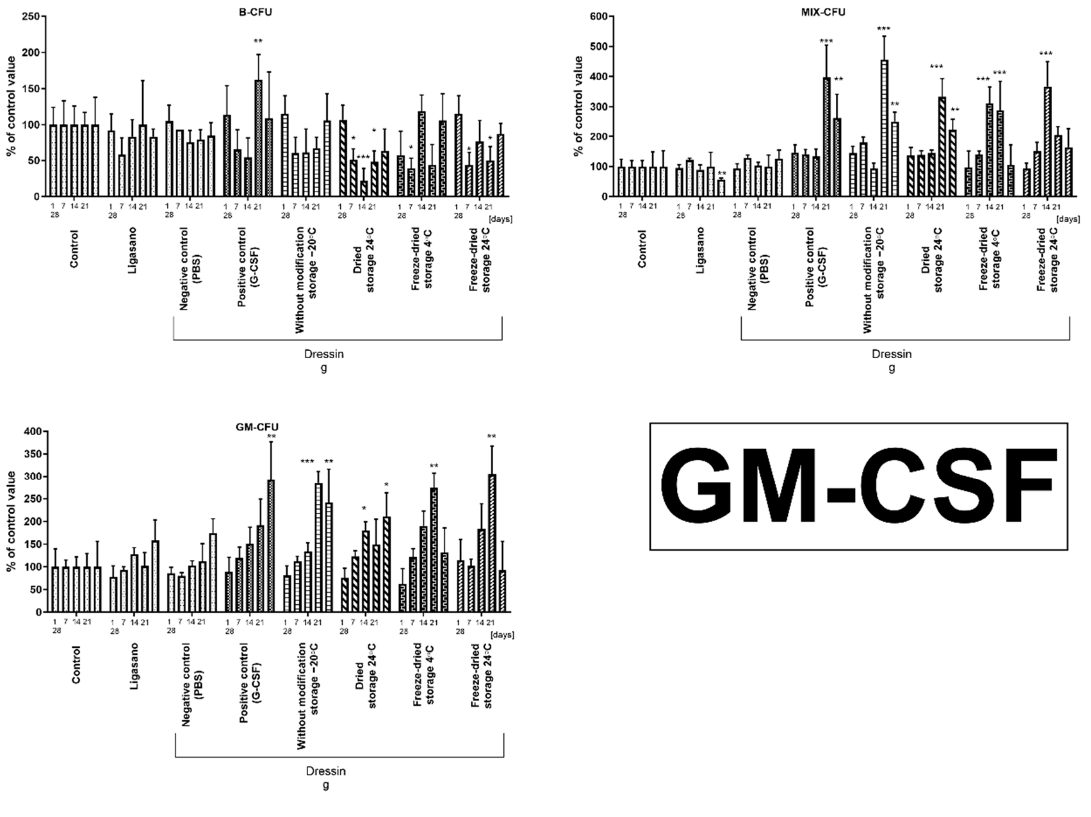

Figure S3 A-B. Evaluation of cytokines activity measured in culture of cord blood hematopoietic stem cells in MethoCult™ H4330 methylcellulose medium with

EPO. A - G-CSF; B - GM-CSF. Cytokines were incorporated into dressings which were prepared using different techniques and were stored 1, 7, 14, 21, and 28 days. Results are presented as mean ± standard deviation. n = 9 (n) - number of samples/group. * - $p < 0.05$; ** - $p < 0.01$; *** - $p < 0.001$ comparisons was made between an appropriate day in experimental and control group values. Statistical analysis in all groups were performed by non-parametric Kruskal-Wallis with Dunn's multiple comparison test.
